# Cross-cultural adaptation and psychometric properties of the Italian version of the Orthorexia Nervosa Inventory (ONI)

**DOI:** 10.1186/s40337-023-00858-0

**Published:** 2023-08-24

**Authors:** Andrea Zagaria, Claudio Barbaranelli, Edoardo Mocini, Caterina Lombardo

**Affiliations:** 1 Department of Psychology, Sapienza University of Rome, Rome, Italy; 2 Department of Experimental Medicine, Sapienza University of Rome, Rome, Italy

**Keywords:** Orthorexia, Psychometric properties, Validation study, Healthy eating, Eating disorders

## Abstract

**Background:**

Orthorexia Nervosa (ON) is an emerging clinical condition characterized by a pathological fixation with healthy eating. Recently, the Orthorexia Nervosa Inventory (ONI) has emerged as a promising tool for assessing orthorexic tendencies and behaviours, aiming to overcome the well-established limitations of existing measures for ON. The present study aimed to examine the psychometric properties of the Italian version of the ONI.

**Methods:**

A total of 879 participants (M_age_ = 33.22 years, SD = 9.19; 56.9% females) completed the ONI along with the Düsseldorf Orthorexia Scale (DOS), measures of disordered eating, obsessive-compulsive symptoms (OCD), and psychosocial impairment. To establish the factorial validity of the ONI, a competing measurement modeling approach was employed by comparing standard confirmatory factor analytic (CFA) with exploratory structural equation modeling (ESEM) solutions. Model-based omega coefficients were computed to examine the internal consistency of the scale. Factorial invariance tests across gender were conducted within a multi-group framework.

**Results:**

A three-factor first-order ESEM solution provided the best and most parsimonious representation of the data: χ^2^(207) = 558.641, p < .001, RMSEA = 0.044 (90% CI 0.040–0.048), CFI = 0.976, TLI = 0.968, SRMR = 0.036. The three latent dimensions, labelled *behaviors*, *emotions*, and *impairments*, showed excellent internal consistency (ω > 0.88). Furthermore, ONI scores were found to be positively correlated with DOS scores, disordered eating, OCD symptoms, and psychosocial impairment, supporting its convergent and criterion validity. Eventually, the ONI was factorially invariant across gender.

**Conclusions:**

Overall, the present study provides evidence for the satisfactory psychometric properties of the ONI in the Italian context, endorsing its use in both clinical and research settings.

**Supplementary Information:**

The online version contains supplementary material available at 10.1186/s40337-023-00858-0.

## Abstract (plain language summary)

Orthorexia Nervosa (ON) is a clinical condition characterized by a pathological fixation with healthy eating. The Orthorexia Nervosa Inventory (ONI) is a recently developed instrument to assess ON behaviours and tendencies. However, the scale has not yet been adapted for the Italian cultural context. To fill this gap, the present investigation aimed to translate the ONI into Italian and examine its psychometric properties on a community sample. The study involved 879 participants (M_age_ = 33.22 years, SD = 9.19; 56.9% females) who completed a series of self-report questionnaires. Findings revealed that the ONI items reflect  three latent dimensions: behaviours and preoccupation with healthy eating; physical and psychosocial impairments; and emotional distress. Importantly, the present study accrued substantial evidence for the internal consistency and criterion validity of the scale, the latter supported by significant correlations with measures of disordered eating, obsessive-compulsive symptoms, and psychosocial impairment. Overall, these findings endorse the use of the Italian version of the ONI for clinical and research practices.

**Supplementary Information:**

The online version contains supplementary material available at 10.1186/s40337-023-00858-0.

## Introduction

The neologism Orthorexia Nervosa (ON) derives from the Greek “ortho” (right, proper) and “orexia” (appetite) and was first introduced by physician Steven Bratman in 1997 to describe a pathological fixation with healthy eating observed among his patients [1]. Despite the growing research interest, a consensus definition of ON has not been established yet [2]. To address this knowledge gap, a multidisciplinary cohort of 47 eating disorders experts from 14 different countries recently conducted a consensus conference, through which they converged to define ON as a mental health disorder that closely relates to the DSM-5 category of “Feeding and Eating Disorders” (F&ED) [3]. Individuals with ON exhibit a strong preoccupation with the quality and nutritional composition of the food, which leads them to impose rigid and inflexible dietary rules and spend an excessive amount of time planning, obtaining, preparing, and eating their food [3, 4]. Compared to anorexia nervosa (AN), where the focus is on the quantity of food intake [5], ON is primarily focused on the quality of food and is sustained by a desire to achieve a feeling of purity and healthiness and by overvalued ideas regarding the health benefits of food [5, 6]. Critically, ON may result in several negative consequences, such as marked emotional distress in relation to food deemed as unhealthy, impaired educational, work, or social life domains, reduced quality of life, and nutritional deficiencies [3, 7].

Despite the growing body of research, the assessment of ON remains challenging due to the limitations and poor psychometric properties of currently available assessment tools [6]. Recently, Opitz and colleagues [8] reviewed all ON-related questionnaires and identified several weaknesses (see also [6]). Notably, the internal consistency of these questionnaires sometimes falls below acceptable levels, discouraging their use. For instance, the reliability of the most employed measure assessing ON, the ORTO-15 [9], has ranged from unacceptable 0.14 to good 0.84 internal consistency values across 24 studies [8]. Moreover, the dimensionality of these questionnaires often remains matter of debate, exhibiting inadequate fit when attempting to reproduce the original factorial structure and showing items to be not relevant to the ON latent dimension [8, 10]. Finally, as noted by Oberle and colleagues [10], there is a lack of specific and comprehensive items assessing emotional distress due to violations of orthorexic dietary rules and physical impairment due to nutritional deficiencies, thereby hindering evaluation. For instance, both the Düsseldorf Orthorexia Scale [11] and the Teruel Orthorexia Scale [12] lack a latent dimension specifically designed for measuring the impairment due to orthorexic symptoms. In light of these challenges, the Orthorexia Nervosa Inventory (ONI) [10] has emerged as a promising new questionnaire for assessing ON, integrating items from established measures and novel items to capture a more comprehensive range of features characterising the clinical condition. The ONI is a 24-item self-report questionnaire composed of three latent dimensions: behaviours and preoccupation with healthy eating; physical and psychosocial impairments; and emotional distress. The original validation study demonstrated strong levels of internal consistency (Cronbach’s alpha of 0.94 for total score and > 0.88 for subscales) and test-retest reliability (r > .86) [10]. Moreover, ONI scores showed significant correlations with disordered eating and obsessive-compulsive symptoms, supporting its concurrent validity [10].

Given the promising evidence regarding the reliability and validity of the ONI, the present study aimed to adapt the instrument for use in the Italian context and investigate its psychometric properties. Specifically, the first aim was to examine the factorial structure of the ONI by comparing standard confirmatory factor analytic models with exploratory structural equation modeling solutions. The second aim was to examine the internal consistency of the scale through model-based omega coefficients. The third aim was to test the convergent and criterion validity of the ONI by examining its relationships with alternative measures of ON, disordered eating, obsessive-compulsive symptoms, and psychosocial impairment. The fourth aim was to test the factorial invariance of the scale across gender.

## Methods

### Procedure

Data were collected via a cross-sectional online survey hosted by Qualtrics (https://www.qualtrics.com). Participants were recruited via word of mouth and advertising on the main social network platforms in February and March 2023. After signing informed consent, participants voluntarily completed the survey without any incentive or compensation. A comprehensive overview of the study was provided on the first page of the survey, and respondents were informed that they could withdraw from the survey at any time by refraining from submitting the form. The online survey lasted about 15 min. The study ensured complete anonymity, with no personally identifiable information collected. The inclusion criteria were being of legal age (18 years or older), being a native Italian speaker, and having given informed consent. Exclusion criteria included participants who were unable to complete the questionnaire due to visual or cognitive impairments. The study design was approved by the Institutional Review Board of the Department of Psychology at Sapienza University of Rome (prot. 0000520; 18/03/2022).

### Participants

The sample size calculation was performed based on three criteria. Firstly, we conducted an RMSEA-based power calculation for the test of close fit on the less parsimonious factorial structure [13]. For a power of 0.80, fixing the critical alpha to 0.05, the minimum sample size needed was 88 (df = 186). Secondly, to detect a practically significant effect size in correlation analyses (ρ = 0.2, [14]), the minimum sample size needed was 193 (power = 0.80; alpha = 0.05, two-tailed test). Thirdly, at least 5 observations per free parameter and 10 observations per each item of the scale were guaranteed according to common rule of thumbs [15, 16].

A total of 879 participants voluntarily completed the survey and were included in the present investigation. The sample comprised 56.9% women, 36.2% men, 4.9% individuals identifying as other or non-binary, and 2% who preferred not to disclose their gender. The mean age of the sample was 33.22 years (SD = 9.19), ranging from 18 to 68. In terms of marital status, 46% of participants were in a relationship, 31.5% were single, 20% were married, 2% were divorced, and 0.5% were widowed. Regarding education, 34% of participants held a master’s degree, 30% held a high-school diploma, 21.7% held a bachelor’s degree, 12.5% held a postgraduate degree, 1.6% held a middle-school diploma, and 0.1% held an elementary school diploma. The mean body mass index (BMI), calculated through self-reported height and weight, was 25.10 kg/m^2^ (SD = 5.37).

### Measures

#### Orthorexia nervosa inventory (ONI)

The Orthorexia Nervosa Inventory (ONI) [10] is a self-report questionnaire that consists of 24 items measuring three latent dimensions: behaviours and preoccupation with healthy eating; physical and psychosocial impairments; and emotional distress. Items are rated on a 4-point scale, ranging from 1 (not at all) to 4 (very). Subscale scores can be computed by summing the items pertaining to each dimension, as well as a total score by adding up each item score. Total scores range from 24 to 96, with higher scores indicating greater orthorexic symptomatology. Table 1 provides an overview of the ONI along with item-level descriptive statistics in the current sample.

**Table 1:** Item-level descriptive statistics

Item	Mean (SD)	Median (IQR)	Min-Max
1. I feel much guilt or self-loathing when I stray from my healthy diet	2.24 (0.84)	2 (1)	1–4
2. I care much more about the healthiness of what I eat than the pleasurable taste of food	1.88 (0.77)	2 (1)	1–4
3. As a result of the amount of time I devote to my healthy diet, I have spent less time than I used to with my family or friends	1.30 (0.59)	1 (0)	1–4
4. I follow a health-food diet rigidly, only eating what my diet allows and not allowing myself any deviations from this diet	1.39 (0.66)	1 (1)	1–4
5. My food restrictions have led me to lose more weight than people would say is good for me	1.23 (0.63)	1 (0)	1–4
6. Preparing food in the most healthful way is very important in my diet	2.44 (0.85)	2 (1)	1–4
7. My healthy eating is a significant source of stress in my relationships	1.42 (0.75)	1 (1)	1–4
8. Over time, my diet has come to include elimination of entire food groups that I believe are unhealthy	1.76 (0.89)	2 (1)	1–4
9. When I stray from my healthy diet, I can only think about what a failure I am	1.67 (0.90)	1 (1)	1–4
10. Health professionals have expressed concern that my diet is too restrictive	1.11 (0.43)	1 (0)	1–4
11. I follow a healthy diet with many rules	1.69 (0.72)	2 (1)	1–4
12. My cleanses or fasts have become more frequent or severe over time	1.12 (0.46)	1 (0)	1–4
13. Whenever I eat anything unhealthy, I feel a great sense of personal impurity	1.74 (0.87)	2 (1)	1–4
14. Even though I have eaten much healthier over time, my physical health has actually declined	1.36 (0.71)	1 (1)	1–4
15. Healthy eating is among the most important things in my life	2.00 (0.81)	2 (1)	1–4
16. As a result of the amount of time I devote to my healthy diet, I have either missed time at work or missed classes at school	1.14 (0.41)	1 (0)	1–4
17. I either do not buy processed food products or I compulsively check the nutrition labels to ensure that only healthy and pure ingredients are included	1.69 (0.76)	2 (1)	1–4
18. The number of healthy dietary rules that I follow has progressively increased over time	1.70 (0.78)	2 (1)	1–4
19. Whenever I feel sick, family or friends comment that the illness may be because my diet is too restrictive	1.18 (0.51)	1 (0)	1–4
20. While spending time with family or friends, I am frequently distracted by thoughts of eating healthily	1.33 (0.68)	1 (0)	1–4
21. Just the thought of me eating something unhealthy makes me very anxious	1.39 (0.70)	1 (1)	1–4
22. I strictly avoid all foods I feel are unhealthy	1.40 (0.64)	1 (1)	1–4
23. Feeling good about my body is completely dependent on me strictly following my healthy diet	1.72 (0.81)	2 (1)	1–4
24. The stricter I become with my diet, the more I seem to experience one or more physical symptoms such as fatigue, faintness, heart racing, nausea, diarrhea, pain, etc.	1.29 (0.63)	1 (0)	1–4

ONI, Orthorexia Nervosa Inventory; SD, standard deviation; IQR, interquartile range; Min, minimum; Max, maximum

#### Cross-cultural adaptation of the Orthorexia Nervosa Inventory (ONI)

After obtaining permission from the authors of the ONI to translate and culturally adapt the questionnaire, we adhered to established guidelines and conducted a forward and back-translation procedure [17]. Firstly, the ONI was independently translated from English into Italian by two individuals with fluency in both languages and expertise in the field of orthorexia. The translations were then compared with each other and with the original English version. Any discrepancies were resolved through consensus, and the two versions were synthesised to produce one common Italian translation. Secondly, a bilingual individual, who was blinded to the original version, back-translated the Italian version of the ONI into English, and the back-translation was further compared with the original version to identify and resolve any semantic inconsistencies. The process was then reviewed by the principal investigator, and a prefinal version was obtained. As a last step, to ensure the clarity, comprehensibility, and lack of ambiguity of the items, the Italian version was administered in a pilot study on a sample of 20 participants from the target population. No further adjustments were deemed necessary. The Italian version of the ONI is available in Additional file 1: Document S1.

#### Düsseldorf Orthorexia Scale (DOS)

In order to assess the convergent validity of the ONI, the Düsseldorf Orthorexia Scale (DOS) [11] was administered. The DOS is a 10-item self-report questionnaire designed to measure orthorexic behaviours and attitudes. Items are rated on a 4-point scale, ranging from 1 (this does not apply to me) to 4 (this applies to me). Total scores range from 10 to 40, with higher scores indicating more severe orthorexic symptoms. The DOS showed satisfactory psychometric properties in the Italian context [18]. Cronbach’s alpha in the present sample was 0.858.

#### Obsessive-Compulsive Inventory-Revised (OCI-R)

In order to evaluate the criterion validity of the ONI, the Obsessive-Compulsive Inventory-Revised (OCI-R) [19] was employed. The OCI-R is a widely used 18-item self-report questionnaire assessing the level of distress stemming from obsessive-compulsive symptomatology. Each item is rated on a 5-point scale, ranging from 0 (not at all) to 4 (extremely). A total score can be calculated by summing the scores for all items (0–72), with higher scores indicating higher severity of obsessive-compulsive symptoms. The OCI-R demonstrated satisfactory psychometric properties in the Italian context [20]. Cronbach’s alpha in the present sample was 0.895.

#### SCOFF questionnaire

The SCOFF questionnaire [21] was employed to address the criterion validity of the ONI. The SCOFF is a brief tool for assessing the core features of Anorexia Nervosa (AN) and Bulimia Nervosa (BN). The questionnaire consists of five dichotomous questions (i.e., 0 = no, 1 = yes), with a total score ranging from 0 to 5 that can be computed based on the sum of affirmative responses. Previous studies (e.g., [22, 23]) have shown that the SCOFF questionnaire is a sensible and specific screening tool for detecting eating disorders (ED). Moreover, the SCOFF showed satisfactory psychometric properties in the Italian context [24].

#### Clinical Impairment Assessment (CIA)

The Clinical Impairment Assessment (CIA version 3.0) [25] was administered to evaluate the criterion validity of the ONI. The CIA is a self-report measure consisting of 16 items designed to evaluate the psychosocial impairment due to eating disorders features over the preceding 28-day period. Each item is rated on a 4-point scale ranging from 0 (not at all) to 3 (a lot). By summing the scores of all items, a total CIA score can be calculated, with a range from 0 to 48. The higher the score, the higher the severity of the impairment. The CIA demonstrated satisfactory psychometric properties in the Italian context [26]. Cronbach’s alpha in the present sample was 0.947.

### Data analytic strategy

Data were analysed using IBM SPSS v. 25 (IBM Corporation, Armonk NY; USA), FACTOR [27], and Mplus v. 8.6 [28].

To examine the dimensionality of the ONI, a three-factor solution was suggested for investigation based on the results of a parallel analysis [29]. Therefore, a preliminary exploratory factor analysis (EFA) was fitted to the inter-item polychoric correlations using the robust weighted least squares—means and variance adjusted (WLSMV) estimator coupled with geomin rotation. The pattern of factor loadings identified in this EFA was consistent with the original validation study conducted by Oberle and colleagues [10], with one exception. Specifically, Item #20, which was originally associated with the “Impairments” latent dimension, exhibited a main loading on the “Emotions” factor.

Accordingly, to establish the factorial validity of the ONI, in line with recent recommendations for the assessment of construct-relevant psychometric multidimensionality, a competing measurement modelling approach was employed by comparing standard confirmatory factor analytic (CFA) with exploratory structural equation modelling (ESEM) solutions (see [30–32]). This approach allows accounting for two relevant sources of multidimensionality [30]: (1) the restrictive and fallible assumption that items are pure indicators of the intended constructs, as they can exhibit non-negligible associations with conceptually related dimensions other than the ones they are meant to measure; (2) the hierarchically-organized nature of the constructs.

To this end, five different models were tested [30]:

1. A unidimensional CFA model positing an overall ON latent factor.
2. A first-order CFA model, in line with the original validation study conducted by Oberle and colleagues [10], positing three correlated latent factors: behaviours, impairments, and emotions. Each item was specified to load on the factor it was supposed to measure, with all cross-loadings fixed at zero.
3. A bifactor CFA model positing a global ON factor and three specific factors: behaviours, impairments, and emotions. All items were allowed to load on the global factor, and each specific factor was reflected by the corresponding items with all cross-loadings fixed at zero. The general and specific factors were specified as orthogonal consistently with standard bifactor assumptions [30].
4. A first-order ESEM model positing the aforementioned three latent factors using a target oblique rotation. Unlike the restrictive independent cluster model of CFA, where cross-loadings are fixed at zero, ESEM with target rotation allows for the estimation of cross-loadings (i.e., non-target loadings) while constraining them to be as close to zero as possible. Through the pre-specification of target and non-target loadings, ESEM provides a more flexible confirmatory approach maintaning an a-priori control on the hypothesised factor structure (e.g., [30, 33])
5. A bifactor ESEM model positing a global ON factor and the aforementioned three specific factors via an orthogonal target rotation. Contrary to the bifactor CFA, cross-loadings on the specific factors were allowed but targeted to be as close to zero as possible.

It should be noted that second-order CFA and ESEM models were not estimated to avoid redundancy, as they are mathematically equivalent to the corresponding three-factor first-order CFA and ESEM solutions [34]. Moreover, since the ONI response format includes four ordered categories and the indicators showed non-negligible deviations from the univariate normal distribution (skewness and kurtosis > |1|), items were treated as ordinal and the factor models were fitted to the inter-item polychoric correlations using the robust weighted least squares—means and variance adjusted (WLSMV) estimator [28].

Given the sensitivity of the chi-square test to large sample sizes and in accordance with a multifaceted approach to the assessment of model fit [35], the following goodness-of-fit indices were reported [36–38]: Root Mean Square Error of Approximation (RMSEA; values ≤ 0.08 indicating reasonable fit and ≤ 0.05 indicating close fit), Comparative Fit Index and Tucker-Lewis Index (CFI and TLI, respectively; values ≥ 0.90 indicating acceptable fit and ≥ 0.95 indicating good fit), and Standardized Root Mean Squared Residual (SRMR; with values of ≤ 0.08 indicating good fit). To determine the best-fitting model, the competing CFA and ESEM models were compared by examining differences in goodness-of-fit indices (e.g., [30–32]). Specifically, differences in CFI and TLI of less than 0.010, in conjunction with differences in RMSEA of less than 0.015, suggest that the more parsimonious model should be preferred [32, 38]. Comparison of goodness-of-fit indices was integrated with a detailed examination of parameter estimates (e.g., [39]), such as target and non-target loadings, and factor correlations. With respect to the size of standardized factor loadings, a value of at least 0.32 (i.e., 10% of overlapping variance) was assumed as a reasonable threshold for interpretative purposes [40].

Internal consistency was assessed through model-based omega coefficients: ω = (Σ | λ_i_ |)^2^ / ([Σ|λ_i_|]^2^ + Σδ_ii_), where λ_i_ denotes the factor loadings and δ_ii_ denotes the uniquenesses [39, 41]. Compared with the traditional alpha coefficient, which relies on the strict assumption of essential tau-equivalence, omega takes into account the strength of association between the items and the latent factors and is suitable under the more realistic congeneric model (see [42]). Moreover, since the ONI was intended to yield a total score in addition to subscale scores, the global reliability index for multidimensional scales was calculated [43]. According to common recommendations, internal consistency values greater than 0.70 are considered acceptable in non-exploratory research [e.g., 44].

Furthermore, a zero-order correlation was calculated with DOS scores to assess the convergent validity of the ONI, while criterion validity was evaluated through zero-order correlations with SCOFF, OCI-R and CIA scores, and BMI. Following Cohen’s [45] benchmarks, correlation coefficients of 0.1 to 0.3 were considered small, 0.3 to 0.5 were considered moderate, and greater than 0.5 were considered large.

Finally, factorial invariance tests across gender were conducted within the stepwise framework proposed by Meredith [46] and adapted for ordered-categorical indicators [47, 48]. Namely, we specified a series of nested models through a multi-group approach: (1) configural invariance (i.e., the same number of factors and same pattern of loadings); (2) metric invariance (i.e., equivalence of factor loadings); (3) scalar invariance (i.e., equivalence of factor loadings and items’ thresholds). To compare these nested models and evaluate the feasibility of the invariance constraints, since χ^2^ was particularly sensitive to large sample sizes, differences in CFI (ΔCFI), TLI (ΔTLI) and RMSEA (ΔRMSEA) were calculated. Specifically, deteriorations greater than 0.010 in CFI and TLI, in conjunction with RMSEA changes higher than 0.015, were considered as a signal for lack of invariance [49, 50].

## Results

### Factor structure

Table 2 presents an overview of the goodness-of-fit indices for the five competing models. Findings revealed that the first-order ESEM provided the best and most parsimonious representation of the data. Although the bifactor ESEM exhibited a slightly better fit, the difference with the first-order ESEM was negligible and the latter was preferred for the sake of parsimony (ΔCFI = 0.005; ΔTLI = 0.004; ΔRMSEA = 0.003). However, as noted by Sánchez-Oliva and colleagues [51] (see also [39]), relying solely on the comparison of goodness-of-fit indices is not sufficient for model selection, and the choice should be complemented with a detailed examination of parameter estimates. By focusing on models’ parameters, two of the three specific factors in the bifactor ESEM were found to be poorly defined, with several target loadings falling below the thresholds for interpretability (i.e., at least 10% of overlapping variance; [40]). Moreover, the comparison of the first-order ESEM with the first-order CFA model revealed that the former exhibited markedly lower inter-factor correlations (r = .474 to 0.641) compared to the latter (r = .668 to 0.800), thus providing a better differentiation between conceptually related latent dimensions [30]. This was consistent with the presence of some non-target cross-loadings, further reinforcing the superiority of the ESEM approach, which acknowledges that items may not function as pure indicators of their respective latent dimensions, as assumed by the restrictive independent cluster model of CFA [30]. Under these conditions, ESEM can be especially advantageous in revealing sources of misfit that would otherwise remain concealed in traditional CFA (e.g., [52]).

**Table 2:** Goodness-of-fit statistics for the five competing models

Model	χ^2^ (df)	CFI	TLI	RMSEA (90% CI)	SRMR
Unidimensional CFA	2174.447 (252)	0.867	0.855	0.093 (0.090–0.097)	0.087
First-order CFA	1060.574 (249)	0.944	0.938	0.061 (0.057–0.065)	0.061
Bifactor CFA	765.358 (228)	0.963	0.955	0.052 (0.048–0.056)	0.049
**First-order ESEM**	**558.641 (207)**	**0.976**	**0.968**	**0.044 (0.040–0.048)**	**0.036**
Bifactor ESEM	456.843 (186)	0.981	0.972	0.041 (0.036–0.045)	0.033

The retained model is highlighted in bold

WLSMV was employed as the parameter estimation method

The retained first-order ESEM showed a great fit to the data: χ^2^(207) = 558.641, p < .001; RMSEA = 0.044 (90% CI 0.040–0.048), p(RMSEA < 0.05) = 0.988; CFI = 0.976, TLI = 0.968, SRMR = 0.036. Parameter estimates are reported in Table 3. Consistent with the hypothesised theoretical dimensions, all factors demonstrated substantial and significant target loadings, indicating that much of the variance was accounted for by the common factors (λ = 0.32–0.92; M = 0.65; *ps* < 0.001). Most of the cross-loadings were lower than 0.30, and the ratio between the primary loading and the cross-loading was higher than 2. The only exception was found for items #7 and #23, which displayed moderate cross-loadings. The occurrence and magnitude of the cross-loadings are in line with the findings from the original validation study of the scale [10] and are also consistent with theoretical reasons discussed in the following section. Moreover, the intercorrelations between the latent factors were found to be moderate-to-large in magnitude. Specifically, the correlation between behaviours and impairments was r = .613, p < .001, the correlation between behaviours and emotions was r = .471, p < .001, and the correlation between emotions and impairments was r = .641, p < .001.

**Table 3:** Standardized parameter estimates from the first-order ESEM model

Item	Standardized factor loadings (λ)			Uniquenesses (δ)
	Factor 1: Behaviours	Factor 2: Impairments	Factor 3: Emotions	
ONI#17	**0.769**	−0.152	0.017	0.520
ONI#11	**0.614**	0.262	−0.051	0.402
ONI#8	**0.427**	0.236	0.126	0.534
ONI#6	**0.846**	−0.176	−0.025	0.450
ONI#22	**0.676**	0.090	0.092	0.382
ONI#18	**0.543**	0.045	0.154	0.562
ONI#15	**0.766**	−0.146	0.101	0.464
ONI#4	**0.679**	0.194	−0.077	0.403
ONI#2	**0.438**	0.179	0.121	0.589
ONI#10	−0.061	**0.906**	0.031	0.209
ONI#24	−0.076	**0.566**	0.224	0.529
ONI#12	−0.128	**0.490**	0.243	0.639
ONI#5	0.060	**0.836**	−0.221	0.436
ONI#19	0.060	**0.834**	−0.140	0.377
ONI#14	0.030	**0.315**	0.282	0.687
ONI#3	0.207	**0.637**	0.011	0.378
ONI#7	−0.005	**0.502**	0.373	0.374
ONI#16	0.283	**0.509**	−0.005	0.489
ONI#9	−0.148	0.054	**0.918**	0.208
ONI#13	0.029	0.066	**0.798**	0.265
ONI#1	0.038	−0.086	**0.896**	0.260
ONI#23	0.366	−0.070	**0.518**	0.491
ONI#21	0.178	0.223	**0.606**	0.227
ONI#20	0.134	0.293	**0.540**	0.286

Factor loadings on the theoretical factors are highlighted in bold

ONI, Orthorexia Nervosa Inventory

Eventually, the unidimensional CFA model revealed a poor fit to the data, χ^2^(252) = 2174.447, p < .001; RMSEA = 0.093 (90% CI 0.090–0.097), p(RMSEA < 0.05) < 0.001; CFI = 0.867, TLI = 0.855, SRMR = 0.087, indicating the absence of substantial common-method variance and supporting the discriminant validity of the posited factors.

### Reliability and validity

Omega coefficients were excellent for all three latent dimensions: Behaviors (ω = 0.885); Impairments (ω = 0.884); Emotions (ω = 0.913). Moreover, a satisfactory global reliability index for multidimensional scales of 0.948 [43] provided psychometric justification for employing a total score to measure ON.

The pattern of zero-order correlations between ONI and the other theoretically related constructs supported the convergent and criterion validity of the scale (see Table 4). Specifically, ONI scores were significantly correlated with DOS scores (r range = 0.667 to 0.876, ps < 0.001), CIA scores (r range = 0.341 to 0.757, ps < 0.001), SCOFF scores (r range = 0.313 to 0.584, ps < 0.001) and OCI-R scores (r range = 0.256 to 0.423, ps < 0.001), with most of these associations showing moderate-to-large magnitudes. Regarding BMI, non-significant associations were found, except for the small correlation observed with the ONI Emotions subscale (r = .180, p < .001).

**Table 4:** Descriptive statistics and zero-order correlations for the ONI dimensions

Dimension	Descriptive statistics	Zero-order correlations				
	Mean (SD)	DOS	OCI-R	SCOFF	CIA	BMI
ONI_Total	37.16 (10.05)	0.876*	0.395*	0.519*	0.643*	0.049
ONI_Behaviors	15.94 (4.71)	0.766*	0.256*	0.313*	0.341*	− 0.040
ONI_Impairments	11.13 (3.27)	0.667*	0.342*	0.456*	0.597*	0.001
ONI_Emotions	10.09 (3.79)	0.786*	0.423*	0.584*	0.757*	0.180*

* p < .001.

ONI, Orthorexia Nervosa Inventory; DOS, Düsseldorf Orthorexia Scale; OCI-R, Obsessive-Compulsive Inventory-Revised; CIA, Clinical Impairment Assessment; BMI, Body Mass Index; SD, standard deviation

### Factorial invariance tests

Factorial invariance tests across gender (males vs. females) were conducted on the three-factor first-order ESEM solution. Participants who identified as other/non-binary could not be included due to the limited sample size. The goodness-of-fit indices were excellent at each step of the analysis. Specifically, the model fitted well when simultaneously tested on both males and females (i.e., configural invariance): χ^2^(414) = 704.796, p < .001; RMSEA = 0.041 (90% CI 0.036–0.047), p(RMSEA < 0.05) = 0.997; CFI = 0.979, TLI = 0.972, SRMR = 0.045. Furthermore, when equality constraints were imposed on factor loadings (i.e., metric invariance) and items’ thresholds (i.e., scalar invariance), there was an improvement in CFI, TLI, and RMSEA. The three levels of gender invariance were thus established, as summarised in Table 5. After demonstrating the adequacy of the scalar invariance model, latent mean differences were examined. The latent means of the female group were freely estimated and scaled on the latent means of the male group. Scores on Behaviours (SMD = 0.059, p = .631) and Impairments (SMD = 0.047, p = .569) were not significantly different across gender. Nonetheless, the latent mean for Emotions was significantly higher in the female group (SMD = 0.414, p < .001).

**Table 5:** Tests of measurement invariance of the ONI across gender

Model	χ^2^ (df)	CFI	TLI	RMSEA (90% CI)	SRMR	Model comparison	ΔCFI	ΔTLI	ΔRMSEA
1. Configural invariance	704.796 (414)	0.979	0.972	0.041 (0.036–0.047)	0.045				
2. Metric invariance	745.588 (477)	0.981	0.977	0.037 (0.032–0.042)	0.051	2 vs. 1	0.002	0.005	-0.004
3. Scalar invariance	772.060 (522)	0.982	0.981	0.034 (0.029–0.039)	0.053	3 vs. 2	0.001	0.004	-0.003

WLSMV was employed as the parameter estimation method

## Discussion

The purpose of the present study was to explore the psychometric properties of the ONI in an Italian community sample, by offering valuable insights into its factorial structure, internal consistency, and convergent and criterion validity.

As a primary step, we investigated the factorial validity of the ONI by employing a competing measurement modelling approach, comparing standard confirmatory factor analytic (CFA) with exploratory structural equation modelling (ESEM) solutions. This allowed us to account for two important sources of psychometric multidimensionality, as highlighted by Morin and colleagues [30]. Firstly, the assumption underlying the traditional independent-clusters-CFA (ICM-CFA) that items serve as pure indicators of the intended constructs is questionable, as they may represent non-pure indicators that exhibit remarkable associations with conceptually related dimensions other than the ones they are primarily assumed to measure [30]. Under this view, ESEM allows for the estimation of non-target cross-loadings while maintaining a substantial confirmatory framework [33]. Secondly, when evaluating the latent structure of a multidimensional scale, the observed indicators might reflect hierarchically-organized constructs [30]. To address this complexity, a comparison of alternative models which includes second-order or bi-factor solutions is warranted in order to accurately reflect the true population model.

Findings revealed that the first-order three-factor ESEM solution offered a satisfactory and parsimonious representation of the underlying structure of the ONI. Moreover, the ESEM solution was factorially invariant across gender, confirming that males and females used a similar frame of reference and the same metric in responding to the ONI items. The three latent factors and the pattern of loadings were almost consistent with those found by Oberle and colleagues [10]. The first factor, named *“behaviours”*, measures behaviours and preoccupation related to healthy eating. The second factor, named *“emotions”*, measures emotional distress resulting from violations of imposed dietary rules. The third factor, named *“impairments*”, measures physical and psychosocial impairments caused by nutritional deficiencies and dietary restrictions. Importantly, all standardized factor loadings were equal to or higher than 0.32, suggesting that a substantial amount of variance in the observed indicators was explained by the latent dimensions [40]. The majority of cross-loadings were observed to be below the threshold of 0.30, and the primary loading-to-cross-loading ratio exceeded 2. However, items #7 and #23 exhibited a cross-loading higher than 0.30 representing an exception to the overall pattern. This may be explained by theoretical reasons. First, item #7 *(“My healthy eating is a significant source of stress in my relationships”)* demonstrated noteworthy loadings on both the “emotions” and “impairments” factors. This finding is consistent with the notion that stress, which may be defined as a negative emotional experience [53], can elicit a range of physiological, behavioural, and emotional responses [54] that may indeed result in both emotional distress and physical impairments. Second, item #23 *(“Feeling good about my body is completely dependent on me strictly following my healthy diet”)* showed meaningful loadings on both the “behaviours” and “emotions” factors. This may be attributed to the fact that the item formulation contains both a behavioural aspect, with the statement *“strictly following my healthy diet”*, and a focus on body dissatisfaction, which has been associated with orthorexic tendencies and experiencing emotional distress [55, 56].

The excellent omega coefficients obtained for the three dimensions of the ONI indicate a high degree of consistency among the items measuring each construct. This finding is further supported by the satisfactory global reliability index for multidimensional scales, which justifies the use of a total composite score to measure ON. These results are in line with the original validation study of the ONI [10], which also demonstrated strong internal consistency with Cronbach’s alpha values of 0.94 and greater than 0.88 for the total score and subscales, respectively. Nevertheless, compared to traditional Cronbach’s alpha, the use of model-based composite reliability coefficients provides several advantages. That is, alpha coefficients may lead to inconsistent estimates of the population internal consistency when the scales do not adhere to tau equivalence, relying on the stringent assumption that all indicators have equal true-score variance [57]. In contrast, composite reliability coefficients are calculated from factor-analytic parameters and are therefore unbiased with varying factor loadings. Furthermore, while Cronbach’s alpha assumes that items reflect a unidimensional structure, composite reliability coefficients can be extended to assess the reliability of multidimensional scales [42]. This flexibility is particularly advantageous as it allows for a more comprehensive assessment of the psychometric properties of a measure, accounting for the multidimensionality of the construct of interest. Consequently, several researchers have advocated for the abandonment of alpha as a reliability measure and for the adoption of alternative model-based estimates (e.g., [57, 58]).

Importantly, the ONI showed very large correlations with DOS scores (r > .667), providing reasonable evidence of convergent validity, i.e., the degree to which two different questionnaires developed to assess a common construct demonstrate substantial and significant correlations [59]. Moreover, the present study offers compelling evidence for the criterion validity of the scale, as demonstrated by the moderate to large zero-order correlations between ONI and measures of disordered eating, obsessive-compulsive symptoms, and impairment secondary to eating disorders features. Consistent with previous meta-analytic findings [60], the associations found between ON and disordered eating may suggest a partial overlap between ON and currently recognized F&ED. As Cena and colleagues [2] have suggested, ON shares core features with the broad field of F&ED, such as the prominent role of food in an individual’s life, heightened concerns about food, dietary restriction, and social and health consequences (e.g., social isolation and malnutrition). Similarly, zero-order correlations found with obsessive-compulsive symptoms are consistent with previous meta-analytic results [60]. These associations may reflect shared features between ON and such symptomatology, including the presence of obsessions and compulsions related to healthy food, as well as ritualistic behaviour related to meal purchase, preparation, and consumption [2]. However, the smaller magnitude of the correlation compared to that with eating disorder symptoms suggests that ON might be conceptualized as a distinct F&ED, rather than belonging to the OCD spectrum, as previously hypothesised [3, 60, 61]. Lastly, non-significant or small correlations were found with BMI, aligning with the majority of previous research in Italian [18], Hungarian [62], Turkish [63], Spanish [12], Greek [64], and Australian [65] samples, which suggested a lack of significant association between ON tendencies and BMI.

Several limitations of the present study should be acknowledged. Firstly, test-retest reliability was not assessed, which precluded us to draw conclusions about the stability of ONI scores over time. Secondly, the sampling method relied on snowball and social media advertising, which may have led to a non-representative sample of the general population. Thirdly, the questionnaires were administered using an online survey platform. Future studies are warranted to investigate the psychometric properties of the ONI using a paper-and-pencil administration. Fourthly, we enrolled a non-clinical sample. Future investigations are needed to administer the ONI in clinical populations with the aim of identifying an optimal cutoff score using receiver operating characteristic (ROC) analysis for discriminating between profiles at risk and not at risk of ON.

## Conclusions

In conclusion, the present study shed light on the good psychometric properties of the ONI in the Italian context, endorsing its use in both clinical and research practices. As highlighted by a recent consensus conference involving a multidisciplinary cohort of 47 ON experts [3], the development of empirically supported assessment tools is imperative to investigate the prevalence of the condition, enhance identification and screening efforts, refine treatment protocols, and ensure appropriate care for affected individuals. In this view, ONI can support clinicians in the identification and treatment of individuals experiencing such conditions. Moreover, the application of the ONI in research settings can significantly contribute to the ongoing efforts to conceptualize this emerging clinical condition, particularly concerning its potential inclusion in the nosographic classifications.

### Supplementary Information

**Additional file 1.** The Italian version of the Orthorexia Nervosa Inventory.

## Data Availability

The datasets used and/or analysed during the current study are available from the corresponding author upon reasonable request.
