# Correction: Cross-cultural adaptation and psychometric properties of the Italian version of the Orthorexia Nervosa Inventory (ONI)

**DOI:** 10.1186/s40337-023-00889-7

**Published:** 2023-09-18

**Authors:** Andrea Zagaria, Claudio Barbaranelli, Edoardo Mocini, Caterina Lombardo

**Affiliations:** 1 Department of Psychology, Sapienza University of Rome, Rome, Italy; 2 Department of Experimental Medicine, Sapienza University of Rome, Rome, Italy

**Correction: Journal of Eating Disorders (2023) 11:144** **https://doi.org/10.1186/s40337-023-00858-0**

The original publication [1] of this article contained an error in the supplement. The error concerns the response scale of the instrument, specifically: “da 0 = Per niente a 4 = Molto”, where it should have been “da 1 = Per niente a 4 = Molto.”

The original article [1] has been corrected.
