# Supplementary material for: Cross-cultural adaptation and psychometric properties of the Italian version of the Orthorexia Nervosa Inventory (ONI)
Source: J Eat Disord. 2023 Aug 24;11:144. doi: 10.1186/s40337-023-00858-0 (PMC10463941; doi:10.1186/s40337-023-00858-0)
Supplement: Supplementary file 1 — Additional file 1. The Italian version of the Orthorexia Nervosa Inventory. [file 40337_2023_858_MOESM1_ESM.pdf]

The Italian version of the Orthorexia Nervosa Inventory (ONI)

Zagaria, A., Barbaranelli, C., Mocini, E., & Lombardo, C. (2023). Cross-cultural adaptation and psychometric properties of the Italian version of the Orthorexia Nervosa Inventory (ONI). Journal of eating disorders, 11(1), 144. https://doi.org/10.1186/s40337-023-00858-0

ISTRUZIONI:

Basandoti sulle tue attuali abitudini alimentari, **indica quanto le seguenti affermazioni siano veritiere per te**, utilizzando una scala di risposta da 1 = Per niente a 4 = Molto.

1. Provo un forte senso di colpa o di disgusto verso me stesso/a quando mi allontano da un'alimentazione sana.
2. Mi preoccupo molto di più della salubrità di ciò che mangio che del gusto piacevole del cibo.
3. A causa del tempo che dedico alla mia alimentazione sana, ho trascorso meno tempo con la mia famiglia o con gli amici rispetto al passato.
4. Seguo rigidamente un'alimentazione sana, mangiando solamente ciò che la mia dieta mi consente e non concedendomi alcuna deviazione da questo regime alimentare.
5. Le restrizioni alimentari mi hanno portato a perdere più peso di quanto le persone ritengono sia salutare per me.
6. Preparare il cibo nella maniera più sana possibile è molto importante nella mia alimentazione.
7. Il mio regime alimentare salutare è una significativa fonte di stress nelle mie relazioni.
8. Con il passare del tempo, nella mia dieta ho eliminato intere categorie di cibi che ritengo non salutari.
9. Quando mi discosto da un'alimentazione sana, riesco solamente a pensare a quanto io sia un fallimento.
10. Professionisti della salute hanno espresso preoccupazioni rispetto alla mia alimentazione troppo restrittiva
11. Seguo un'alimentazione sana con molte regole.
12. I miei digiuni purificatori sono diventati più frequenti o intensi con il passare del tempo.
13. Ogni volta che mangio qualcosa di poco salutare, provo una forte sensazione di impurità.

14. Nonostante abbia mangiato in modo più salutare nel corso del tempo, la mia salute fisica è in realtà peggiorata.
15. Mangiare sano rientra tra le cose più importanti nella mia vita.
16. A causa del tempo che dedico alla mia alimentazione sana, ho dovuto tralasciare il lavoro o saltare lezioni a scuola.
17. Non compro cibi trattati e industriali, o controllo ripetutamente le etichette nutrizionali per accertarmi che siano presenti solamente ingredienti sani e naturali.
18. Il numero di regole che seguo per un'alimentazione salutare è aumentato progressivamente nel corso del tempo.
19. Ogni volta che mi sento male, familiari o amici pensano che la malattia potrebbe essere causata dalla mia dieta troppo restrittiva.
20. Mentre trascorro del tempo in famiglia o con amici, sono spesso distratto da pensieri riguardanti il mangiare sano
21. Il solo pensiero di me che mangio qualcosa di non salutare mi rende ansioso.
22. Evito rigorosamente tutti i cibi che non ritengo sani.
23. Sentirmi bene con il mio corpo dipende interamente dal seguire rigorosamente un’alimentazione sana
24. Più divento rigido/a con la mia dieta, più mi sembra di sperimentare uno o più sintomi fisici come stanchezza, svenimenti, tachicardia, nausea, diarrea, dolori, etc.

SCORING:

Behaviors: 2+4+6+8+11+15+17+18+22

Impairments: 3+5+7+10+12+14+16+19+24

Emotions: 1+9+13+20+21+23
